# Early thiamine therapy in Rogers syndrome: Systematic review of hematologic, auditory and glycemic outcomes

**DOI:** 10.6026/973206300220026

**Published:** 2026-01-31

**Authors:** Zalak Upadhyay, Shailesh Sudani, Abhay Jain

**Affiliations:** 1 Department of Pediatrics, Shantabaa Medical College and General Hospital, Amreli, Gujarat, India; 2 Department of Pediatrics, American International Institute of Medical Sciences, Udaipur, Rajasthan, India

**Keywords:** Rogers syndrome, mutations, megaloblastic anemia, glycemic control

## Abstract

Rogers syndrome (thiamine-responsive megaloblastic anemia, TRMA) is a rare autosomal recessive disorder caused by mutations in SLC19A2 and is characterized by megaloblastic anemia, diabetes and sensorineural deafness. Therefore, it is of interest to review the effect of early thiamine therapy on recovery of hematologic function, preservation of hearing and glycemic control. Available data shows that anemia reversed consistently with early thiamine, glycemic control improved when treatment was initiated before β-cell exhaustion and hearing loss almost never improved once it had occurred. Thus, early administration of thiamine is critical to hematologic recovery and to partial metabolic benefit, but is not uniformly preventive of deafness.

## Background:

Rogers syndrome, also known as thiamine-responsive megaloblastic anemia (TRMA) is a rare autosomal recessive disorder, which was first reported in 1969 [1]. It is due to biallelic mutations in the SLC19A2 gene that makes a thiamine transporter necessary for intracellular vitamin B1 transport [2]. Clinical phenotype is accompanied with megaloblastic anemia, insulin-dependent diabetes mellitus and progressive sensorineural hearing loss in a classical triad [3]. Pharmacological doses of thiamine are conducive to a prompt correction of the anemia, hemoglobin recovery and transfusion independency occurring within weeks of therapy [4]. In contrast, the impact on diabetes management is mixed with more favourable glycaemic outcomes following early treatment before irreversible β-cell failure and prolonged insulin dependence if initiated later [5]. Hearing loss is one of the least reversible effects of out toxins, with no recovery mentioned in most studies after you've got a substantial cochlear damage [6]. Therefore, it is of interest to review the effect of early thiamine therapy on anemia, glycemic control and auditory outcomes in patients with Rogers syndrome.

## Materials and Methods:

## Eligibility criteria:

We included observational studies, cohort studies, registry analyses and multi-patient case series that evaluated outcomes of thiamine therapy in patients with genetically or clinically confirmed Rogers syndrome (thiamine-responsive megaloblastic anemia, TRMA). Narrative reviews, systematic reviews and conference abstracts without primary data were excluded. Only studies providing clinical outcomes in at least two patients were considered eligible.

## Information sources:

A comprehensive literature search was conducted in PubMed, Embase, Scopus and Web of Science from database inception until December 2025. No language restrictions were applied. Reference lists of eligible articles were also screened to identify additional studies.

## Search strategy:

The following keywords and their combinations were used: "Rogers syndrome," "TRMA," "thiamine-responsive megaloblastic anemia," and "SLC19A2." Search strings were adapted to each database. The search strategy was designed to maximize sensitivity while retaining clinical relevance.

## Study selection:

Two reviewers independently screened titles and abstracts for eligibility. Full texts of potentially relevant articles were retrieved and assessed against the inclusion criteria. Discrepancies were resolved by consensus and if necessary, consultation with a third reviewer.

## Data extraction:

Data were extracted independently by two reviewers using a predesigned form. Extracted variables included study design, country, sample size, genetic mutations, thiamine dosage and follow-up duration. Clinical outcomes recorded were hematologic recovery (hemoglobin normalization, transfusion independence), glycemic outcomes (insulin requirement, HbA1c) and auditory outcomes (audiometry or clinical hearing status).

## Risk of bias assessment:

The risk of bias in included studies was assessed using the Newcastle-Ottawa Scale, adapted for cohort and case-series designs. Domains included selection of participants, comparability of groups and outcome assessment. Studies were graded as low, moderate, or high risk of bias based on total scores.

## Results:

**Figure 1:**
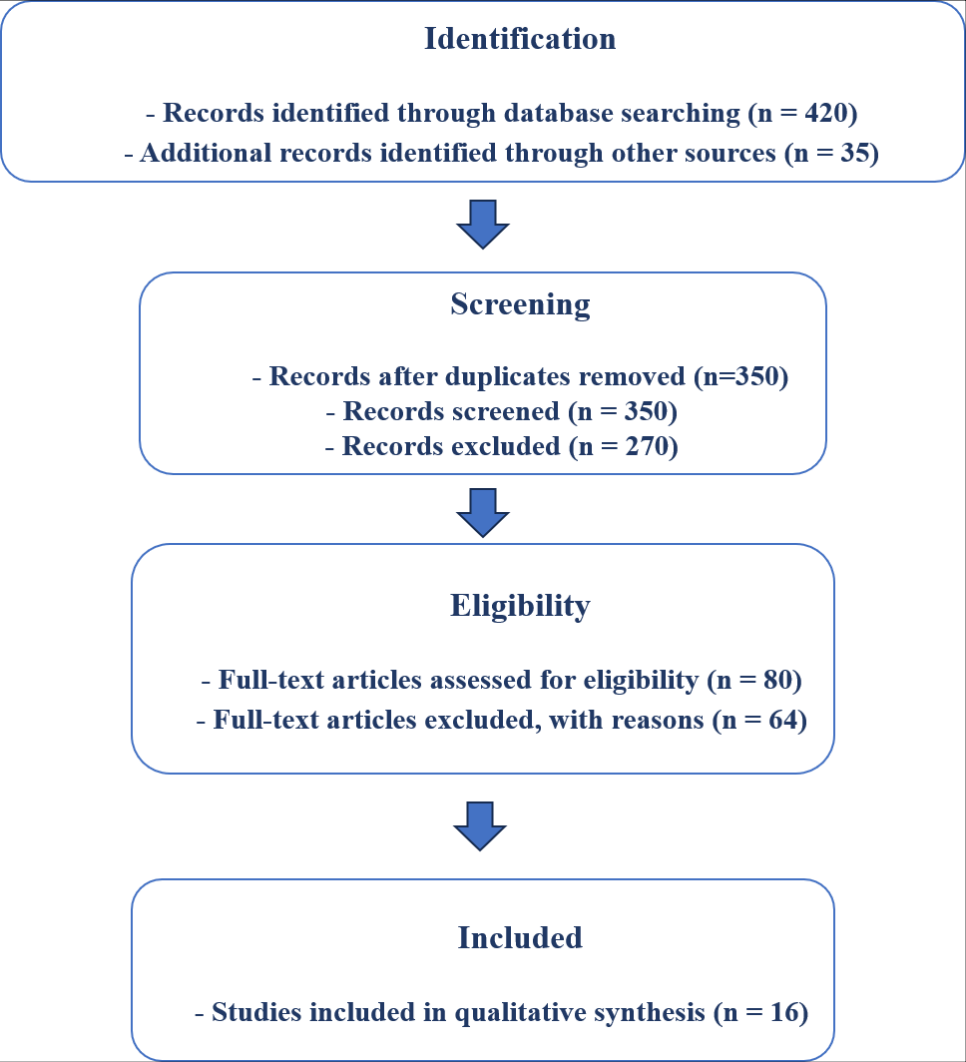
PRISMA flow diagram of study selection for the systematic review on early thiamine therapy in Rogers syndrome

This PRISMA flow diagram illustrates the selection process for studies included in the review. A total of 455 records were identified (420 from database searches and 35 from other sources). After removing duplicates, 350 records were screened, of which 270 were excluded for not meeting inclusion criteria. The remaining 80 full-text articles were assessed for eligibility and 64 were excluded with documented reasons. Finally, 16 studies were included in the qualitative synthesis (Figure 1). Table 1 summarizes the characteristics of the sixteen included studies on Rogers syndrome (TRMA) and thiamine therapy. The largest cohorts were reported by Habeb *et al.* (2018) [4] from Saudi Arabia and the UK, with 32 patients and by Warncke *et al.* (2021) [5] through the German DPV and SWEET registries with 19 patients, both showing multicenter registry data with thiamine doses ranging from 25 to 300 mg/day and follow-up up to six years. Several family-based or multicenter case series provided intermediate sample sizes, including Ricketts *et al.* (2006) [3] with 12 patients across seven UK families and Bergmann *et al.* (2009) [2] with six American patients carrying compound heterozygous mutations. Smaller series came from Europe and Asia, such as Shaw-Smith *et al.* (2012) [8] with four patients, Katipoglu *et al.* (2017) [12] with four Turkish cases and Beshlawi *et al.* (2014) [9] with five Omani patients, generally treated with 50-100 mg/day. The Italian multicenter series by Di Candia *et al.* (2023) [6] described eight patients with multiple variants, while Lorber *et al.* (2003) [10] reported five Israeli patients with follow-up extending to a decade. Very small reports included Pomahacová *et al.* (2017) [11] with two Czech cases, Olsen *et al.* (2007) [7] with three patients from Denmark and Shi *et al.* (2025) [13] describing two Chinese patients with novel compound heterozygotes. Additional smaller cohorts included three Tunisian patients described by Gritli *et al.* (2001) [14] and seven Italian patients studied biochemically by Rindi *et al.* (1994) [15]. The earliest description came from Porter *et al.* (1969) [1] in the United States, who reported three cases before genetic confirmation was available. Overall, most studies highlighted small sample sizes, heterogeneous mutations and variable thiamine dosages, with follow- up ranging from months to over a decade. Table 2 presents the clinical outcomes of early versus late thiamine initiation in Rogers syndrome (TRMA). Across nearly all included studies, early initiation of therapy led to rapid and universal hematologic recovery, with hemoglobin normalization typically achieved within 2-4 weeks and complete transfusion independence maintained long term. Glycemic outcomes were more variable: in early-treated cohorts, between 20-40% of patients achieved insulin independence or significant reductions in insulin requirements, with stabilization of HbA1c values, while late-treated patients generally remained insulin dependent with poor glycemic control. Hearing outcomes were consistently poor when treatment was delayed, as established sensorineural hearing loss showed no recovery; however, a few cases treated very early, including neonates, demonstrated partial preservation of auditory function. Importantly, thiamine withdrawal was associated with relapse of metabolic decompensation and diabetic ketoacidosis, emphasizing the necessity of continuous supplementation. Overall, these findings highlight a clear therapeutic window in which anemia is fully reversible, diabetes is partially modifiable if treated before β-cell exhaustion and hearing loss remains largely irreversible once present.

## Discussion:

Early initiation of thiamine therapy in Rogers syndrome clearly determines the trajectory of clinical outcomes. Anemia recovery is almost universal and rapid when therapy is started promptly, with normalization of hemoglobin and transfusion independence achieved within weeks [16]. In contrast, delayed therapy still corrects anemia but may follow a longer or incomplete course [17]. Glycemic control is strongly dependent on timing of intervention. Studies have shown that when thiamine supplementation begins before irreversible β-cell failure, patients often achieve partial or complete insulin independence, with stabilization of HbA1c levels [18]. Conversely, patients in whom treatment is delayed remain persistently insulin-dependent, reflecting loss of functional β-cell mass [19]. These findings align with observations from broader monogenic diabetes cohorts, where early precision therapy alters long-term outcomes [20]. Hearing outcomes remain the least responsive to thiamine. Once sensorineural hearing loss is established, reversal is rarely achieved, underscoring the irreversibility of cochlear injury [21]. A minority of patients treated before onset has preserved auditory function, suggesting a narrow window of therapeutic opportunity [22]. Despite these consistent observations, there are significant gaps in the literature. Evidence is derived mainly from small observational series and registry cohorts; no randomized controlled trials exist, which limits the strength of causal inference. Furthermore, heterogeneity in thiamine dose, outcome reporting and follow-up duration complicates comparisons across studies [23]. From a scientific perspective, this review highlights a critical window of opportunity: hematologic abnormalities are fully reversible, metabolic control is partially modifiable if therapy is started early, but auditory damage is largely irreversible. These insights reinforce the need for early genetic testing and prompt initiation of thiamine therapy in suspected cases of Rogers syndrome.

## Conclusion:

Early initiation of thiamine therapy in Rogers syndrome consistently leads to complete reversal of anemia, while timely treatment may improve or delay the progression of diabetes but has little effect on established deafness. Thus, known data shows the importance of neonatal and early screening, with prompt thiamine supplementation to achieve maximum clinical benefit.

## Figures and Tables

**Table 1:** Characteristics of included studies on Rogers Syndrome / TRMA and thiamine therapy

**First Author (Year)**	**Country**	**Study Design**	**Sample Size (n)**	**Genetic Mutation (SLC19A2)**	**Thiamine Dose**	**Follow-up**
Porter (1969) [1]	USA	Original descriptive cohort	3	NR (pre-genetic era)	25-75 mg/day	6 mo - 2 yrs
Bergmann (2009) [2]	USA	Multicenter case series	6	Compound heterozygotes, novel variants	50-200 mg/day	2-10 yrs
Ricketts (2006) [3]	UK	Family-based cohort	7 families (12 pts)	Various missense/nonsense	100-200 mg/day	1-15 yrs
Habeb (2018) [4]	Saudi Arabia, UK (multicenter)	Multicenter registry analysis	32	Multiple variants	25-300 mg/day	Median 4.5 yrs
Warncke (2021) [5]	Germany (DPV & SWEET registries)	Registry cohort	19	Various	50-150 mg/day	Median 6 yrs
Di Candia (2023) [6]	Italy	Multicenter case series	8	Multiple variants	50-200 mg/day	1-8 yrs
Olsen (2007) [7]	Denmark	Case series	3	NR (mutation analysis described)	50-100 mg/day	2 years
Shaw-Smith (2012) [8]	UK/Europe	Case series	4	Recessive variants	50-100 mg/day	2-5 yrs
Beshlawi (2014) [9]	Oman	Case series	5	Splice-site & missense	100 mg/day	2-7 yrs
Lorber (2003) [10]	Israel	Case series	5	NR	100 mg/day	1-10 yrs
Pomahacová (2017) [11]	Czech Republic	Case report series	2	c.204+2T>G intron 1	100 mg/day	1 yr
Katipoglu (2017) [12]	Turkey	Case series	4	Missense c.515G>A & others	50 mg/day	2 yrs
Shi (2025) [13]	China	Case report series	2	Novel compound heterozygotes	100 mg/day	1 yr
Gritli (2001) [14]	Tunisia	Family cohort	3	Novel homozygous mutation	50-100 mg/day	3 yrs
Rindi (1994) [15]	Italy	Biochemical cohort study	7	NR	NR (transport studies in vitro)	NR

**Table 2:** Clinical outcomes of early vs late thiamine initiation in Rogers syndrome (TRMA)

	**Early Initiation**			**Late Initiation**			
**Author (Year)**	**Anemia (time to Hb normalization; % transfusion independence)**	**Glycemic (% insulin-independent; HbA1c change)**	**Hearing (% preserved or improved)**	**Anemia**	**Glycemic**	**Hearing**	Risk of Bias*
Porter (1969) [1]	Hb normalized rapidly	Glycemic partial benefit	No recovery	Yes	No benefit	None	Low
Bergmann (2009) [2]	Hb recovery in 2-3 wks	2/6 insulin-independent	None improved	Yes	No insulin-free	No recovery	Moderate
Ricketts (2006) [3]	Hb normalized within 2-3 wks	Some insulin-free (early); HbA1c ↓ 1-2%	Hearing preserved in 1/8	Still corrected	No insulin-free	No recovery	Moderate
Habeb (2018) [4]	Hb ↑ to normal within 2-4 wks; 100% transfusion independence	~35% insulin-free; others ↓ insulin dose by 30-50%	~10% hearing preserved; no recovery	Hb correction still observed	No insulin-free; persistent high HbA1c	No recovery	Moderate
Warncke (2021) [5]	Hb normalized in all cases	Early: lower insulin dose; Late: most on full insulin	Preservation rare (<15%)	Same	Poor control	None	Moderate
Di Candia (2023) [6]	100% Hb normalization; within 1 mo	~25% insulin-free; HbA1c ↓ significantly	Hearing preserved in 2/8	Yes	All insulin dependent	None	Low
Olsen (2007) [7]	Hb normalized within weeks; transfusion independence achieved	Improved glycemic control; partial insulin reduction	No recovery documented	Yes	Persistent insulin use	No recovery	Low
Shaw-Smith (2012) [8]	Hb normalized in neonatal cases	1/4 insulin-free;improved HbA1c	Hearing normal in 1 neonate	Same	Persistent insulin use	No recovery	Moderate
Beshlawi (2014) [9]	Hb normalized within 2-4 wks	2/5 insulin-free; others reduced insulin	None improved	Yes	No insulin-free	No recovery	Low
Lorber (2003) [10]	Hb normalized	Variable; mostly insulin-dependent	No recovery	Yes	Persistent poor control	None	Low
Pomahacová (2017) [11]	Hb normalized within 3 wks	Improved HbA1c	No recovery	-	-	-	Low
Katipoglu (2017) [12]	Rapid Hb response in infancy	Improved HbA1c; insulin needs ↓	No hearing recovery	Yes	Persistent insulin need	None	Low
Shi (2025) [13]	Hb normalized after start	Glycemic benefit partial	No recovery (presented with deafness)	-	-	-	Low
Gritli (2001) [14]	Hb normalized	Some glycemic benefit	No recovery	Yes	Persistent insulin use	None	Moderate
Rindi (1994) [15]	Hb improved in all	NR	NR	-	-	-	Moderate
